# Influence of body size and skeletal maturity status on strength and motor performances of soccer players 9–16 years

**DOI:** 10.1038/s41598-024-55042-4

**Published:** 2024-02-27

**Authors:** Jan M. Konarski, Mateusz Skrzypczak, Duarte Freitas, Robert M. Malina

**Affiliations:** 1 Theory of Sports Department, Poznań University of Physical Education, Królowej Jadwigi 27/39, Poznań, Poland; 2 Department of Physical Education and Sport, University of Madeira, Funchal, Portugal; 3 Centre of Research, Education, Innovation, and Intervention in Sport (CIFI2D), Faculty of Sport, University of Porto, Porto, Portugal; 4 Department of Kinesiology and Health Education, University of Texas at Austin, Austin, TX USA; 5 School of Public Health and Information Sciences, University of Louisville, Louisville, KY USA

**Keywords:** Youth sports, Skeletal age, Speed, Acceleration, Agility, Endurance, Medical research, Paediatric research

## Abstract

The contributions of height, weight and skeletal age (SA) to strength and motor performances of male soccer players 9–12 (n = 60) and 13–16 (n = 52) years were estimated. SA was assessed with the Fels method, and was expressed as the standardized residual of the regression of SA on chronological age CA (SAsr). Static strength (right + left grip), speed (5 m, 20 m sprints), acceleration (10 to 20 m), agility (figure-of-eight run), explosive strength (vertical jump) and endurance (yo–yo intermittent shuttle run, 13–16 years only) were measured. Hierarchical multiple regression was used. The interaction of SAsr with body size (height and height x weight interaction) explained most of the variance in strength in both age groups, 9–12 years (51.6%) and 13–16 years (56.7%), and in speed (31.4%, 38.7%), acceleration (39.6%), and explosive strength (32.6%) among players 13–16 years. In contrast, SAsr alone explained limited amounts of variance in strength, speed, acceleration and vertical jump among players 9–12 years (1.4–4.5%) and 13–16 years (0–0.5%). Results for agility varied with CA group, while SAsr per se was the primary contributor to endurance among players 13–16 years (18.5% of the variance). Although the influence of body size and skeletal maturity status on performances was significant, the explained variance differed among tasks and between CA groups, and suggested a role for other factors affecting performances of the soccer players.

## Introduction

Studies of the contribution of variation in maturity status to the strength and motor performances of youth athletes, primarily males, vary in design, methods of analysis and results. For example, stage of pubic hair was a significant predictor of jumping and strength among basketball players 12–13 years, and of only jumping performance among players 14–15 years^1,2^. Skeletal age (SA), along with chronological age (CA), height and weight were among significant predictors of several functional capacities and sport-specific skills among soccer players 11–12 and 13–14 years^3^, while stage of pubic hair and height were significant predictors of functional capacities and soccer-specific skills among players 13–15 years^4,5^. And in adolescent soccer players, a series of cross-sectional and longitudinal analyses based on allometry and multilevel modelling considered relationships among SA, pubertal status, body size, estimated body composition and training with several performance tasks (sprinting, agility, vertical jump, endurance shuttle run) and soccer-specific skills (ball control, dribbling speed, shooting accuracy, wall pass); variable relationships among skeletal maturity status, body size and specific performances were noted^6–9^. In the context of the preceding, an issue in studies relating performances of youth to SA and pubertal status is the difficulty in partitioning the influence of maturity status per se from that associated with CA and body size given the interrelationships among CA, maturity status and body size during childhood and adolescence.

In the context of the preceding, the purpose of this study is to estimate the contributions of height, weight and SA to the performances of youth soccer players 9–12 and 13–16 years of age on tests of strength, speed, acceleration, power and cardiovascular endurance. The age groups approximate, respectively, the transition from late childhood into adolescence and adolescence per se. The analysis initially considers the contributions of height and weight to the respective performances, the mediation effects of the relationships among height, weight and SA on each performance task, and then the specific contribution of SA per se to each performance task. It was hypothesized that the influence of SA on strength and motor performance is mediated largely through its effect on height and weight, and that SA per se has a relatively limited influence on performances.

## Methods

The study was approved by the Human Ethics Research Committee of Karol Marcinkowski Medical University in Poznań (No. 186/19) and was in accord with the Declaration of Helsinki. The study was also approved by the soccer club and coaches. Each player and his parents or legal guardians provided written informed consent. The research was conducted by Faculty and staff of the Theory of Sports Department at the Poznań University of Physical Education.

### Sample

The sample included 112 male soccer players of European ancestry, 9.6–16.4 years of age from two soccer clubs in West-Central Poland. The playing season included two stages, mid-September through late November and March through June, and involved 24–30 games evenly divided between in the fall and spring. All players participated in formal training sessions of about 90 min duration on five days per week, and generally in one game per week (Saturday or Sunday) during the 9-month season, except for the winter break, in addition to regular participation in physical education during the school year. Training sessions were held on mixed artificial or natural turf, and included a combination of physical, technical and tactical activities. The duration of games varied among competitive CA groups: U10: 2 × 25 min halves, U12: 2 × 30 min, U14: 2 × 40 min, and U16: 2 × 45 min. Unfortunately, information on the number of matches and specific playing time in each match for individual players was not available.

This study was conducted in early May 2018 after the eighth match in the Spring. The 112 players were divided into two CA groups for analysis, 9–12 years which spans the transition from late childhood into adolescence, and 13–16 years which includes adolescence per se. CA was calculated as the difference between date of testing and date of birth, and expressed as decimal age.

### Body size and composition

All players were measured and tested prior to training on the morning of a single day three days after the last game and one day after recovery training. Height and weight were measured and several performance tests were administered at the facilities of the Poznań University of Physical Education.

Heights and weights were measured under standard conditions by an experienced biological anthropologist, using established procedures^10^. Height was measured with shoes removed to the nearest 0.1 cm using a portable stadiometer [Harpenden, Crosswell, Crymych, Pembrokeshire, UK]. The player was in the standard erect posture with weight evenly distributed between both feet, heels together, arms hanging relaxed as the sides and the head in the Frankfurt horizontal plane. Weight was measured with a Tanita MC-780 scale [Tanita Corporation, Japan] which also provided an estimate of percentage fat, in turn fat-free mass (FFM) and fat mass (FM).

### Maturity status

Radiographs of the left hand and wrist were taken and evaluated with the Fels method to estimate SA and its associated standard error^11^. All radiographs were evaluated by a single individual with considerable experience (RMM). Fels SAs ranged from 7.52 to 14.67 years among players 9–12 years and from 12.64 to 17.98 years among players 13–16 years; no player was skeletally mature.

The difference of SA minus CA was used to classify players in each CA group as average or “on time” (SA within ± 1.0 year of CA), advanced (early, SA in advance of CA by more than + 1.0 year), or delayed (late, SA less than CA by more than -1.0 year) in skeletal maturity status. The band of ± 1.0 year approximates standard deviations for SA within specific CA groups, and allows for error associated with assessments and variation among methods of SA assessment^12,13^.

### Performance tests

Several tests were measured in both CA groups. The players wore appropriate shoes for the testing session which was conducted on a surface designed to accommodate regular organized games and competitions at the Poznań University of Physical Education. Not all players were available for performance testing; as such, numbers varied among tasks.

Grip strength of each hand was measured with a Lafayette dynamometer to the nearest kg (model 78,010, Lafayette Instrument Company, Indiana, USA). While standing erect with the arm at the side and not touching the body and the elbow bent slightly, the player was instructed to give a maximal effort. The test was repeated alternately with each hand three times with a pause of one minute between trials. The best trial with each hand was retained and the sum of grip strength with the right and left hands was used for analysis.

Speed was measured as the time elapsed in single 5 m and 20 m sprints. The former is an indicator of starting speed, while the latter is an indicator of maximal speed of the sprint. The test began with a standing start 0.5 m behind the starting line. Time elapsed from crossing the starting line to the line at 5 m and time elapsed from the crossing starting line to the line at 20 m were recorded to 0.001 s using a digital laser photocell system (Witty, Microgate, Italy). The time at crossing 10 m during the 20 m sprint was also recorded as an estimate of acceleration^14^. Sprints were repeated twice, and the better time for each variable was retained^15^. Accuracy, validity and reliability of the speed and acceleration protocol were established in earlier studies^16^.

Agility was measured using the figure-of-eight run^15,17,18^. The player stood at the starting line midway between two 1.2 m bars placed 5 m apart, and was instructed to run three figure-of-eight laps around the bars as fast as possible. Elapsed time was measured to 0.01 s using the digital lased photocell system (Witty, Microgate, Italy) located at the start/finish line. The better of two trials was retained for analysis.

Explosive strength was measured as the vertical jump. The player stood with one side against the measuring board and reached as high as possible with the arm straight; this height was recorded. Then from a half-squat position with the trunk bent forward, the player jumped upward as high as possible and touched the board at the highest point of the jump. The difference between height of the jump and height of standing reach was the indicator of vertical jump performance measured to the nearest 1.0 cm. Three consecutive jumps were performed; the best was retained for analysis.

Aerobic capacity was measured with the yo-yo intermittent shuttle run, level 1^19^. The test involved running between two markers 20 m apart, following audio cues which dictate the running speed. After each 2 × 20 m run following the tempo prescribed by the audio cues, the participant had a 10 s break during which he walked 2 × 5 m, and then began the next 2 × 20 m shuttle. The running tempo increased at regular intervals, and the test continued until the player was no longer able to maintain the required pace. The total distance covered by the player was recorded in meters. One trial was given.

### Analysis

Descriptive statistics (means and standard deviations) for CA, SA, height, weight, estimated body composition and each performance test were calculated for players 9–12 and 13–16 years. The assumptions of normal distribution and homogeneity of variances were assessed with, respectively, the Kolmogorov–Smirnov statistic and the Levene’s test. One-way ANOVA was used to compare the CA, SA, height, weight, estimated body composition and performances of players in the two CA groups and also of players of contrasting skeletal maturity status within each CA group.

Within each CA group, a three-step hierarchical multiple regression model was used to estimate the contribution of Fels SA to the explained variance in grip strength and each motor performance test after controlling for height and weight, and the interaction of SA with body size. Since SA and CA are highly correlated (r = 0.89 in the total sample), SA was regressed on CA, and the standardized residual (SAsr) was retained as the indicator of skeletal maturity status for the analysis. SAsr provides an estimate of the influence of SA independent of CA^20^.

The correlations between weight and FM, and between weight and FFM > 0.70 in both age groups , and as such do not permit the inclusion of another block of variables or the addition of FFM and FM to the first block. Moreover, the inclusion of FFM and FM in the regression models together with weight will result in multicollinearity which, in turn, leads to imprecise estimates of regression coefficients and large standard errors.

Body weight, the sum of right and left grip strength, 20 m sprint, agility and vertical jump were non-normally distributed. The ladder of powers^21^ including their graphical representation (gladder) was used to identify the appropriate transformation to convert the respective variables into normal distributions. The indicated variables were transformed using inverse, 1/cubic, square, or 1/square root. Height, weight, and SAsr were z-standardized within each CA group to reduce co-linearity. Since the effect of height on strength and motor performance may depend on weight, or the effect of SA on motor performance may depend on height or weight, or both height and weight, interaction terms were created by multiplying height × weight, SAsr × height, SAsr × weight, and SAsr × height × weight.

For grip strength and each motor performance test, independent variables were entered into the hierarchical multiple regression in 3 blocks: Block 1 included height, weight, and height × weight interaction; Block 2 included the interactions of SAsr × height, SAsr × weight, and SAsr × height × weight; and Block 3 included only SAsr. This order of entry was based on the overall results in studies relating SA to performance tasks which highlighted the relationships between body size and the interaction of body size and SA (see Introduction). However, because height and weight were highly correlated (r > 0.70), weight and SAsr × weight were removed from the models due to co-linearity^22^. The final hierarchical multiple regression analyses of the strength and motor variables thus included height and height × weight in block 1; SAsr × height, and SAsr × height × weight in block 2; and SAsr in block 3. The variance inflation factors (VIF) values were ≤ 2.370 for players 9–12 years and ≤ 2.570 for players 13–16 years, indicating an absence of multi-co-linearity in the regression models. All statistical analyses were performed in STATA, version 16 (StataCorp 2019) and IBM SPSS Statistics, version 28.0 (IBMCorp 2019). The level of significance was set at 5%.

## Results

Descriptive statistics for CA, SA, height, weight, and performances of players 9–12 and 13–16 years are summarized in Table 1. As expected, the older players are significantly taller, heavier and stronger, and perform significantly better on the sprints, acceleration, agility and vertical jump than the younger players. Although the differences between groups for the two sprints, acceleration and agility are seemingly small, they are significant.

**Table 1:** Sample sizes and descriptive statistics (mean [M], standard deviation [SD]) for chronological age (CA), skeletal age (SA), height, weight, estimated body composition, and motor performances of soccer players 9–12 and 13–16 years, and results of the analyses of variance comparing the two CA groups.

	9–12 years			13–16 years			F*
	n	M	SD	n	M	SD	
CA, yrs	60	11.3	0.9	52	14.5	1.0	294.05
Fels SA, yrs	60	11.9	1.7	52	15.7	1.3	168.93
Height, cm	60	147.5	8.4	52	168.6	9.0	166.44
Weight, kg	60	38.1	7.0	52	56.6	8.7	154.23
Fat-free mass, kg	60	31.3	5.5	51	47.6	7.4	176.41
Fat mass, kg	60	6.8	1.9	51	8.8	1.8	29.26
Fat mass, %	60	17.8	2.7	51	15.6	2.3	19.72
Grip strength, sum R + L, kg	59	38.1	7.6	51	68.9	13.9	214.03
5 m sprint, sec	58	1.17	0.09	43	1.07	0.07	34.13
20 m sprint, sec	58	3.58	0.21	43	3.13	0.20	119.13
Acceleration, from 10 to 20 m, sec	58	2.04	0.12	43	1.81	0.11	103.44
Agility, sec	57	13.22	0.72	42	12.58	0.49	24.57
Vertical jump, cm	56	36.7	6.5	42	49.8	9.7	63.74
Endurance run, distance, m				34	1526	533	

*All F ratios are significant *p* < 0.001.

### Players of contrasting maturity status

The distributions of players in the two CA groups by skeletal maturity status based on the difference of SA minus CA are summarized in Table 2. The three maturity groups are present among players 9–12 years, but only nine players (15%) are late or delayed in skeletal maturity status, while 26 (43%) are average or on time and 25 (42%) are early or advanced. In contrast, no players 13–16 years are late in skeletal maturity status, while equal numbers (n = 26) are classified as average and early. Within each CA group, differences in CA among players in the respective maturity groups do not significantly differ. Of interest, mean SA is in advance of mean CA by about two years among early maturing players in the two CA groups.

**Table 2:** Sample sizes, mean (M) and standard deviation (SD) for chronological age (CA), skeletal age (SA), height, weight, estimated body composition and motor performances of soccer players of contrasting skeletal maturity status among players 9–12 and 13–16 years.

	Players 9–12 years										Players 13–16 years						
	Skeletal maturity status										Skeletal maturity status^†^						
	Late			Average			Early			F	Average			Early			F
	n	M	SD	n	M	SD	n	M	SD		n	M	SD	n	M	SD	
CA, yrs	9	11.4	0.8	26	11.0	1.0	25	11.5	0.9	1.84	26	14.3	1.0	26	14.6	1.0	1.09
Fels SA, yrs	9	9.6	1.1	26	11.2	1.2	25	13.4	0.9	51.67***	26	14.8	1.0	26	16.5	1.0	36.51***
Height, cm	9	142.6	6.9	26	144.5	6.2	25	152.3	8.6	9.23***	26	164.8	9.3	26	172.4	6.9	11.18**
Weight, kg	9	34.4	4.5	26	35.9	5.3	25	41.8	7.7	7.51***	26	52.0	8.2	26	61.1	6.7	19.04***
Fat-free mass, kg	9	28.8	3.4	26	29.3	3.9	25	34.2	6.2	7.52***	26	44.0	7.2	25	51.3	5.7	15.82***
Fat mass, kg	9	5.6	1.2	26	6.5	1.7	25	7.6	2.1	4.74**	26	8.0	1.6	25	9.6	1.7	11.01**
Fat mass, %	9	16.1	2.0	26	18.0	2.4	25	18.1	3.1	1.96	26	15.4	2.5	25	15.8	2.2	0.22
Grip strength, sum R + L, kg	9	33.7	6.2	26	35.4	5.8	24	42.8	7.4	10.49***	26	62.8	12.7	25	75.3	12.4	12.65***
5 m sprint, sec	8	1.17	0.08	25	1.18	0.10	25	1.17	0.09	0.11	24	1.08	0.08	19	1.06	0.06	1.13
20 m sprint, sec	8	3.52	0.23	25	3.60	0.18	25	3.58	0.23	0.48	24	3.18	0.20	19	3.08	0.18	2.95
Acceleration from 10 to 20 m, sec	8	2.01	0.11	25	2.05	0.11	25	2.05	0.13	0.33	24	1.82	0.11	19	1.78	0.11	1.54
Agility, sec	7	12.8	0.6	25	13.2	0.7	25	13.3	0.7	1.73	23	12.6	0.5	19	12.5	0.5	0.34
Vertical jump, cm	7	40.4	9.4	24	35.3	5.4	25	37.0	6.4	1.80	23	47.7	10.7	19	52.5	7.9	2.65
Endurance run, distance, m											18	1336	495	16	1740	506	5.54*

^†^No players 13–16 years were late in skeletal maturity status; *p < 0.05 **p ≤ 0.01 ***p ≤ 0.001.

Early maturing players 9–12 years are significantly taller and heavier, have a larger FFM, and are stronger than their average and late maturing peers, but players in the latter groups do not differ significantly in height, weight, estimated body composition and grip strength. On the other hand, players 9–12 years in the contrasting skeletal maturity groups do not differ significantly in performances on the sprints, figure-of-eight agility run and the vertical jump and in acceleration.

Early maturing players 13–16 years are also significantly taller and heavier, have a larger FFM and FM, are stronger, and perform better in the endurance run compared to their average maturing peers. But as in the younger CA group, early and average maturing players do not differ significantly in the sprints, acceleration, agility and vertical jump.

### Hierarchical regression analyses

Results of the hierarchical regression analyses among players 9–12 years and 13–16 years are summarized in Table 3. Complete tables of the respective hierarchical multiple regression analyses are available in the Supplementary Tables (Tables S1–S7).

**Table 3:** Results of the hierarchical regression analyses: estimated variance (%) in strength and motor performances of soccer players 9–12 and 13–16 years explained by body size and the standardized residuals of the regression of SA on CA (SAsr).

	9–12 years			13–16 years		
	Block 1	Block 2	Block 3	Block 1	Block 2	Block 3
	*R*^2^	Δ*R*^2^	Δ*R*^2^	*R*^2^	Δ*R*^2^	Δ*R*^2^
Grip strength, sum R + L, kg	51.6***	2.8	1.7	56.7***	0.6	0.0
5 m sprint, sec	8.9	6.0	1.4	31.4***	3.4	0.0
20 m sprint, sec	10.1*	5.3	4.3	38.7***	3.1	0.5
Acceleration, from 10 to 20 m, sec	9.4	5.5	4.5	39.6***	2.0	0.1
Agility, sec	1.0	5.2	12.7**	6.7	14.3*	0.4
Vertical jump, cm	5.6	0.3	4.5	32.6***	1.3	0.4
Endurance run,				11.8	2.0	18.5*

Block 1—variance explained by body size; Block 2—additional variance explained by the interactions between body size and SAsr; and Block 3—variance explained by SAsr alone (Sample sizes are as in Table 1).

Block 1: height and height × weight; Block 2: SAsr × height and SAsr × height × weight; Block 3: SAsr.

*p < 0.05 **p < 0.01 ***p < 0.001.

Among soccer players 9–12 years, height and height × weight (block 1) explain 51.6% of the variance in grip strength (*p* < 0.001) and 10.1% of the variance in the 20 m sprint (*p* < 0.05). The amounts of variance explained in the other performances are not significant and relatively small: 5 m sprint (8.9%), acceleration (9.4%), vertical jump (5.6%) and agility (1.0%). The interactions of SAsr × height and SAsr × height × weight (block 2) account for relatively small and non-significant amounts of additional variance in the 5 m sprint (6.0%), acceleration (5.5%), 20 m sprint (5.3%), agility (5.2%), grip strength (2.8%) and the vertical jump (0.3%). SAsr alone (block 3) accounts for 12.7% of the variance in agility (*p* < 0.01) over and above the variance explained by height and height × weight (block 1) and SAsr × height and SAsr × height × weight interactions (block 2). For the other performances, the variance explained by SAsr alone is small and non-significant: grip strength (1.7%), 5 m sprint (1.4%), 20 m sprint (4.3%), acceleration (4.5%) and vertical jump (4.5%) over and above that explained by body size (block 1) and SAsr-body size interactions (block 2).

In contrast, among players 13–16 years, height and the height × weight interaction (block 1) account for significant (*p* < 0.001) amounts of variance in grip strength (56.7%), acceleration (39.6%), 20 m sprint (38.7%), vertical jump (32.6%) and 5 m sprint (31,4%), and for relatively small, non-significant amounts of variance in the yo-yo endurance run (11.8%) and agility (6.7%). The addition of the interactions of SAsr × height and SAsr × height × weight (block 2) accounts for a significant (*p* < 0.05) amount of additional variance in the agility run (14.3%), but for small and non-significant amounts of additional variance in the 5 m (3.4%) and 20 m (3.1%) sprints, acceleration (2.0%), endurance run (2.0%), the vertical jump (1.3%), and grip strength (0.6%). The variance explained by SAsr alone (block 3) adds significantly (*p* < 0.05) to the explained variance only in the endurance run (18.5%) over and above blocks 1 and 2. However, the variance explained by SAsr alone is negligible and non-significant beyond that explained by blocks 1 and 2 for the 20 sprint (0.5%) and acceleration (0.1%), agility and the vertical jump (both 0.4%), and accounts for none of the variance in grip strength and 5 m sprint among the players 13–16 years.

## Discussion

Results of the hierarchical regression analyses indicated similar results for grip strength in the two CA groups of soccer players. Height and the height × weight interaction (block 1) accounted for a significant proportion of the variance in grip strength, 51% among players 9–12 years and 57% among players 13–16 years (Table 3). Of interest, the interactions of SAsr with height and SAsr with height and weight (block 2), and SAsr per se (block 3) added relatively little to the explained variance in strength over and above that explained by height and the height x weight interaction.

The results of the hierarchical regression analyses also highlighted the importance of body size per se in performances on the sprints, acceleration and vertical jump among players 13–16 compared to players 9–12 years. Among players 13–16 years, height and the height × weight interaction (block 1) explained 31–40% of the variance in speed, acceleration and power, compared to 6–10% of variance among the younger players. The interactions of SAsr with height and SAsr × height × weight (block 2) explained relatively small amounts of additional variance, 0.3% to 6% among players 9–12 years and 1–3% among players 13–16 years. SAsr per se (block 3) explained small amounts of additional variance in speed, acceleration and power among players 9–12 years, 1–5%, and players 13–16 years, 0.0-0.5%.

For the agility expressed in the figure-of-eight run, height and the height × weight interaction (block 1) explained only 1% and 7% of the variance among players 9–12 and 13–16 years, respectively, but the addition of SAsr × height and SAsr × height × weight to the model (block 2) increased the explained variance in agility to 5% among players 9–12 years and 14% among players 13–16 years. Of interest, SAsr by itself (block 3) explained an additional 13% of the variance in agility among the younger players, but < 1% of the variance among the adolescent players. Though limited to players 13–16 years, body size (block 1) and SAsr (block 3) individually explained, respectively, 12% and 19% of the variance in the yo-yo intermittent endurance, while the interaction of SAsr and body size (block 2) explained only 2% of the variance.

The preceding results should be considered in the context of growth in body size per se, maturity-related changes during the interval of adolescence, and individual differences in the timing and tempo of the adolescent growth spurt. Body size is a primary factor that influences strength during childhood, the transition into adolescence and during adolescence. In contrast, motor performances in late childhood and the transition in adolescence are largely influenced by opportunities to use specific skills on a regular basis than body size per se and individual differences in maturity status^23^. In the present sample of soccer players, maturity-related differences in body size and grip strength are clear, allowing for variation in CA among the maturity groups; on the other hand, the contrasting maturity groups of players 9–12 and 13–16 years do not differ in speed, acceleration, agility and the vertical jump (Table 2). During the interval of adolescence, performances are influenced by the differential timing of the adolescent growth spurts in height, weight and performances among individuals. Based on longitudinal observations, body weight, grip strength and the vertical jump have growth spurts that occur, on average, after the growth spurt in height, and aerobic power has an adolescent spurt that occurs, on average, close to age at PHV in both boys and girls, while limited data for running speed assessed with a shuttle run suggest a spurt prior to age at PHV^23,24^. The present study, in contrast, is cross-sectional so that inferences about the differential timing of adolescent spurts in body size and performances need to be addressed with caution.

Studies addressing the influence of body size, maturity status and other variables on the performances of youth soccer players are variable in the age range of players, performance tests and methods of analysis. Using multiple linear regression, skeletal maturity status expressed as SA (Fels method) divided by CA (SACA ratio) was among significant predictors of only performance in the counter-movement jump in soccer players 11–12 and 13–14 years, but was not among predictors of a 35 m slalom sprint, an agility shuttle run, and the yo-yo endurance run in the two age groups of players^3^. Also using multiple linear regression analysis, pubertal status was among significant predictors of performances on a 30 m sprint (along with body weight), the vertical jump (along with height), and the yo-yo intermittent endurance run (along with years of training) among soccer players 13–15 years^4^.

In mixed-longitudinal samples of soccer players spanning 11–17 years, the independent influence of CA and SA (Fels method) along with estimated fat-free mass, fat mass and performances in the yo-yo endurance run and the counter movement jump on performances of repeated sprints (sum of 7, 34.2 m sprints including a slalom with a 25-s rest between sprints) and an agility shuttle run were considered^6,8^. Focus was on modelling performance changes by CA and SA across adolescence. In subsequent studies of speed (repeated sprints) and agility (shuttle run, labelled change of direction) in the mixed-longitudinal samples of soccer players, results of the multi-level modelling of performance varied with skeletal maturity status at 12–14 years, and the difference persisted through adolescence^7,9^.

The preceding studies highlighted the effects of maturity status on performances of early adolescent soccer players and the persistence of maturity-related differences in sprinting speed and agility across adolescence. The studies also highlighted the interactions among SA, CA, estimates of body composition, yo-yo endurance run and counter movement jump. Though interesting and significant, the specific contribution of skeletal maturity status per se to the respective performances, independent of body size or composition, and perhaps other variables, was not considered.

Although not directly comparable with previous research given the characteristics of the samples, method of SA assessment and statistical approaches, results from the present study were generally consistent with two earlier studies which indicated a relatively small influence of SA (TW2 20 bone method^25^) alone or of SA interacting with body size on the strength and several motor performances among males. The predictive value of CA, SA, height and weight, and their interactions to several performance tasks was considered in a mixed-longitudinal sample of Belgian boys 12–19 years^26^. Limiting observations to boys 12–17 years, the explained variance for static strength (arm pull) was highest (36–58%), followed by the vertical jump (4–17%), step test (2–4%), and 50 m sprint/shuttle run (0–3%).

The standardized residual (SAsr) of the regression of SA on CA was the indicator of skeletal maturity status in a cross-sectional study of American boys and girls 7–12 years^20^. Limiting observations to boys 9–10 and 11–12 years of age, SAsr per se or in interaction with height and weight influenced right and left grip strength (27–46%) more than the standing long jump (10%, 13%) and sprint (4%, 19%). Allowing for the different analytical protocols and CA ranges, the estimated explained variances overlapped those in the present study (Table 3).

More recently, hierarchical multiple regression was used in a study of the relationship between skeletal maturity status (TW3 radius, ulna, short bone method^27^ expressed as SAsr and motor coordination in youth 11–14 years^28^. The four tests comprising the Körperkoordinations Test für Kinder (KTK) battery^29^ were administered to 284 boys. After controlling for height and weight, the interactions of SAsr with height and weight accounted for only 0.3–8.7% of the variance in the four coordination tests in single year CA groups 11–14 years. Similarly, after controlling for height and weight and the interactions of SAsr with height and weight, the variation explained by SAsr alone in the individual coordination tests ranged from 0.0 to 8.1%^28^. Overall, the variance in the four motor coordination tasks explained by body size and SAsr overlapped the estimates for speed, acceleration and the vertical jump in the present study (Table 3).

The present study attempted to estimate the unique and incremental contribution of SAsr, over and above body size, to the variance in grip strength and motor performances of male soccer players 9–16 years of age. Results of the present study as well as observations from the literature highlighted the complexity of partitioning the relationships among SAsr, height and weight and the strength and motor performances of youth soccer players. The effects of SAsr on grip strength in the present study were mainly expressed though body size in players of the two age groups considered, 9–12 and 13–16 years. The effects of SAsr on speed, acceleration and power were also significantly expressed through body size among players 13–16 years, while among players 9–12 years the three blocks of the hierarchical multiple regression analyses explained relatively small amounts of the variance in the sprints, acceleration and vertical jump. By inference, other variables that may influence these performances merit attention among players during the interval spanning late childhood and the transition into adolescence. In contrast, results for the figure-of-eight run (agility) were somewhat variable. Among players 9–12 years, SAsr alone accounted for 13% and the interactions of SAsr with height and with height × weight accounted for 5% of the variance in agility, while body size per se had a negligible influence. Among players 13–16 years, on the other hand, the interaction of SAsr with height and with height × weight accounted for 14%, and height and the height × weight interaction accounted for 7% of the variance in the agility run, while SAsr alone had virtually no influence on agility.

## Summary

The effects of SAsr on grip strength were mainly expressed though body size in each age group of soccer players. The effects of SAsr on speed, acceleration and power were also largely expressed through body size among players 13–16 years, while relatively small amounts of the variance in the sprints, acceleration and vertical jump were explained by SAsr interacting with body size in players 9–12 years. The latter thus suggested an important role for other factors affecting performances among the younger players. Results for agility (figure-of-eight run) were also variable within each CA group, while SAsr alone explained a significant percentage of the variance in the endurance run over that explained by body size and the interaction of body size with SA in players 13–16 years. Although explained variances varied among tasks and between CA groups, the results highlight the interactions among CA, skeletal maturity status, height and weight with the strength and motor performances of youth soccer players spanning late childhood-early adolescence and adolescence per se.

### Practical applications

The results have relevance for coaches and others working with youth athletes, specifically in understanding the complexity of relationships among CA, biological maturity status, body size and commonly used tests of fitness and performance among youth in the context of trainability, specialization and selection, and in the decision-making process. The findings may also help athletes in understanding their own strength and motor performances relative to their growth and maturity status, and guide parents in supporting their children participating in sport activities. Nevertheless, the relatively small amount of variance in the performance tests explained by skeletal maturity status per se or in combination with body size among male soccer players highlights the need to consider other factors affecting that may influence the performances of youth athletes. In concert with genotype and neuromuscular maturation per se, other factors include training history, quality of coaching, parental pressures, specific instruction and practice, skills learned through free play and/or physical education classes, and motivation and other psychosocial factors that are beyond the scope of the present study.

### Supplementary Information

Supplementary Tables.

## Data Availability

Correspondence and requests for materials should be addressed to J.M.K.
