# Supplementary material for: Influence of body size and skeletal maturity status on strength and motor performances of soccer players 9–16 years
Source: Sci Rep. 2024 Feb 27;14:4768. doi: 10.1038/s41598-024-55042-4 (PMC10899180; doi:10.1038/s41598-024-55042-4)
Supplement: Supplementary file 1 — Supplementary Tables. [file 41598_2024_55042_MOESM1_ESM.pdf]

Influence of body size and skeletal maturity status on strength and motor performances of soccer players 9-16 years

Jan M. Konarski[1*], Mateusz Skrzypczak[1], Duarte Freitas[2] and Robert M. Malina[3]

Supplementary Tables

Table S1. Hierarchical regression analysis for body size and standardised residuals of Fels skeletal age on chronological age in grip strength (sum of left + right)

	Grip Strength (sum left and right), kg††								
	Step 1			Step 2			Step 3		
Variable	*B*	*SE B*	*β*	*B*	*SE B*	*β*	*B*	*SE B*	*β*
9-12 years (n = 59)									
Height	5,16	0,73	0,68***	5,24	0,90	0,69***	5,13	0,89	0,68***
Height × weight†	-0,74	0,66	-0,11	-1,84	0,89	-0,27*	-1,66	0,89	-0,24
SAsr × height				-1,68	0,92	-0,23	-1,36	0,94	-0,19
SAsr × height × weight				0,08	0,46	0,02	0,46	0,52	0,12
SAsr							1,30	0,91	0,17
R^2		0,516***			0,544			0,560	
ΔR^2					0,028			0,017	
13-16 years (n = 51)									
Height	1446,58	189,55	0,76***	1377,13	216,43	0,72***	1376,58	219,35	0,72***
Height × weight	42,15	163,05	0,03	47,55	171,95	0,03	47,43	173,88	0,03
SAsr × height				19,14	199,04	0,01	17,10	208,94	0,01
SAsr × height × weight				119,62	162,76	0,08	113,94	227,28	0,08
SAsr							10,50	289,50	0,01
R^2		0,567***			0,573			0,573	
ΔR^2					0,006			0,000	

†Inverse transformed; ††square transformed (13-16 years).

B, unstandardized coefficients; *SE B*, standard error of *B*; *β*, standardized coefficients.

ΔR^2, R^2 change; height and weight are standardized estimates; SAsr, standardized residuals of skeletal age on chronological age.

SAsr × height, interaction of SAsr with height; SAsr × height × weight, interaction of SAsr with height and weight; variables entered the model at step 1: height and height × weight; variables entered the model at step 2: SAsr × height, and SAsr × height × weight; variables entered the model at step 3: SAsr.

***p <0.001.

Table S2. Hierarchical regression analysis for body size and standardised residuals of Fels skeletal age on chronological age in the 5 m sprint.

	5 m Sprint, sec								
	Step 1			Step 2			Step 3		
Variable	*B*	*SE B*	*β*	*B*	*SE B*	*β*	*B*	*SE B*	*β*
9-12 years (n = 58)									
Height	-0,03	0,01	-0,31*	-0,04	0,01	-0,48**	-0,05	0,01	-0,49**
Height × weight†	-0,01	0,01	-0,07	-0,01	0,01	-0,15	-0,01	0,01	-0,12
SAsr × height				-0,01	0,02	-0,10	-0,01	0,02	-0,06
SAsr × height × weight				-0,01	0,01	-0,28	-0,01	0,01	-0,19
SAsr							0,01	0,02	0,16
R^2		0,089			0,149			0,163	
ΔR^2					0,060			0,014	
13-16 years (n = 43)									
Height	-0,04	0,01	-0,49***	-0,04	0,01	-0,58***	-0,04	0,01	-0,58***
Height × weight	0,01	0,01	0,16	0,01	0,01	0,17	0,01	0,01	0,17
SAsr × height				0,002	0,01	0,02	0,002	0,01	0,02
SAsr × height × weight				0,01	0,01	0,20	0,01	0,01	0,20
SAsr							0,001	0,02	0,01
R^2		0,314***			0,348			0,348	
ΔR^2					0,034			0,000	

†Inverse transformed.

B, unstandardized coefficients; *SE B*, standard error of *B*; *β*, standardized coefficients.

ΔR^2, R^2 change; height and weight are standardized estimates; SAsr, standardized residuals of skeletal age on chronological age.

SAsr × height, interaction of SAsr with height; SAsr × height × weight, interaction of SAsr with height and weight; variables entered the model at step 1: height and height × weight; variables entered the model at step 2: SAsr × height, and SAsr × height × weight; variables entered the model at step 3: SAsr.

$^{*}p$ <0.05; $^{**}p$ <0.01; $^{***}p$ <0.001.

Table S3. Hierarchical regression analysis for body size and standardised residuals of Fels skeletal age on chronological age in the 20m sprint.

	Sprint 20m, sec††								
	Step 1			Step 2			Step 3		
Variable	*B*	*SE B*	*β*	*B*	*SE B*	*β*	*B*	*SE B*	*β*
9-12 years (n = 58)									
Height	-0,07	0,03	-0,32*	-0,10	0,03	-0,48**	-0,11	0,03	-0,50**
Height × weight†	0,00	0,03	-0,01	-0,02	0,03	-0,08	-0,01	0,03	-0,04
SAsr × height				-0,02	0,03	-0,10	-0,01	0,04	-0,03
SAsr × height × weight				-0,03	0,02	-0,26	-0,01	0,02	-0,11
SAsr							0,06	0,03	0,27
R^2		0,101			0,154			0,197	
ΔR^2					0,053			0,043	
13-16 years (n = 43)									
Height	0,003	0,001	0,59***	0,004	0,001	0,65***	0,004	0,001	0,65***
Height × weight	0,0004	0,001	-0,08	-0,0003	0,001	-0,07	-0,004	0,001	-0,07
SAsr × height				-0,001	0,001	-0,10	-0,001	0,001	-0,12
SAsr × height × weight				-0,001	0,001	-0,15	-0,001	0,001	-0,23
SAsr							0,001	0,001	0,11
R^2		0,387***			0,418			0,423	
ΔR^2					0,031			0,005	

†Inverse transformed; ††1/cubic transformed (13-16 years).

B, unstandardized coefficients; *SE B*, standard error of *B*; *β*, standardized coefficients.

ΔR^2, R^2 change; height and weight are standardized estimates; SAsr, standardized residuals of skeletal age on chronological age.

SAsr × height, interaction of SAsr with height; SAsr × height × weight, interaction of SAsr with height and weight; variables entered the model at step 1: height and height × weight; variables entered the model at step 2: SAsr × height, and SAsr × height × weight; variables entered the model at step 3: SAsr.

* p <0.05; ** p <0.01; *** p <0.001.

Table S4. Hierarchical regression analysis for body size and standardised residuals of Fels skeletal age on chronological age in acceleration from 10 to 20m sprint.

	Acceleration from 10 to 20m, sec								
	Step 1			Step 2			Step 3		
Variable	*B*	*SE B*	*β*	*B*	*SE B*	*β*	*B*	*SE B*	*β*
9-12 years (n = 58)									
Height	-0,04	0,02	-0,30*	-0,05	0,02	-0,44**	-0,06	0,02	-0,46**
Height × weight†	0,00	0,01	0,01	-0,01	0,02	-0,12	-0,01	0,02	-0,08
SAsr × height				-0,02	0,02	-0,18	-0,01	0,02	-0,11
SAsr × height × weight				-0,01	0,01	-0,23	0,00	0,01	-0,07
SAsr							0,03	0,02	0,28
R^2		0,094			0,149			0,194	
ΔR^2					0,055			0,045	
13-16 years (n = 43)									
Height	-0,06	0,01	-0,56***	-0,07	0,02	-0,61***	-0,07	0,02	-0,61***
Height × weight	0,02	0,01	0,16	0,01	0,01	0,14	0,01	0,01	0,15
SAsr × height				0,01	0,02	0,08	0,01	0,02	0,09
SAsr × height × weight				0,01	0,01	0,11	0,01	0,02	0,14
SAsr							-0,004	0,02	-0,04
R^2		0,396***			0,416			0,417	
ΔR^2					0,020			0,001	

†Inverse transformed.

B, unstandardized coefficients; *SE B*, standard error of *B*; *β*, standardized coefficients.

ΔR^2, R^2 change; height and weight are standardized estimates; SAsr, standardized residuals of skeletal age on chronological age.

SAsr × height, interaction of SAsr with height; SAsr × height × weight, interaction of SAsr with height and weight; variables entered the model at step 1: height and height × weight; variables entered the model at step 2: SAsr × height, and SAsr × height × weight; variables entered the model at step 3: SAsr.

*p <0.05; **p <0.01; ***p <0.001.

Table S5. Hierarchical regression analysis for body size and standardised residuals of Fels skeletal age on chronological age in the figure-of-eight run (agility).

	Agility – figure-of-eight run, sec††								
	Step 1			Step 2			Step 3		
Variable	*B*	*SE B*	*β*	*B*	*SE B*	*β*	*B*	*SE B*	*β*
9-12 years (n = 57)									
Height	0,000005	0,000009	0,08	0,000017	0,000012	0,25	0,000020	0,000011	0,29
Height × weight†	-0,000002	0,000009	-0,04	-0,000004	0,000012	-0,06	-0,000008	0,000011	-0,13
SAsr × height				-0,000002	0,000012	-0,03	-0,000010	0,000012	-0,15
SAsr × height × weight				0,000010	0,000006	0,29	0,000001	0,000006	0,02
SAsr							-0,000032	0,000011	-0,47**
R^2		0,010			0,062			0,189	
ΔR^2					0,052			0,127**	
13-16 years (n = 42)									
Height	-0,12	0,08	-0,24	-0,16	0,09	-0,33	-0,16	0,09	-0,32
Height × weight	0,02	0,07	0,05	-0,004	0,07	-0,01	-0,003	0,07	-0,01
SAsr × height				0,15	0,08	0,29	0,16	0,08	0,31
SAsr × height × weight				0,09	0,06	0,24	0,12	0,09	0,31
SAsr							-0,05	0,11	-0,10
R^2		0,067			0,210			0,214	
ΔR^2					0,143*			0,004	

†Inverse transformed; ††1/cubic transformed (9-12 years).

B, unstandardized coefficients; *SE B*, standard error of *B*; *β*, standardized coefficients.

ΔR^2, R^2 change; height and weight are standardized estimates; SAsr, standardized residuals of skeletal age on chronological age.

SAsr × height, interaction of SAsr with height; SAsr × height × weight, interaction of SAsr with height and weight; variables entered the model at step 1: height and height × weight; variables entered the model at step 2: SAsr × height, and SAsr × height × weight; variables entered the model at step 3: SAsr.

**p <0.01; **p <0.001.

Table S6. Hierarchical regression analysis for body size and standardised residuals of Fels skeletal age on chronological age in the vertical jump.

	Vertical jump[††]								
	Step 1			Step 2			Step 3		
Variable	*B*	*SE B*	*β*	*B*	*SE B*	*β*	*B*	*SE B*	*β*
9-12 years (n = 56)									
Height	-0,002	0,002	-0,16	-0,002	0,002	-0,14	-0,0024	0,0025	-0,17
Height × weight[†]	0,002	0,002	0,14	0,001	0,002	0,09	0,0017	0,0025	0,13
SAsr × height				-0,001	0,003	-0,08	-0,0001	0,0026	-0,01
SAsr × height × weight				0,0002	0,0013	0,03	0,0014	0,0014	0,18
SAsr							0,0040	0,0025	0,28
R^2		0,056			0,059			0,104	
ΔR^2					0,003			0,045	
13-16 years (n = 42)									
Height	479,37	122,08	0,54***	506,64	139,66	0,57***	502,28	141,54	0,57**
Height × weight	-60,38	105,01	-0,08	-49,14	110,96	-0,06	-50,07	112,20	-0,07
SAsr × height				-72,58	128,43	-0,08	-88,61	134,82	-0,10
SAsr × height × weight				-53,70	105,02	-0,08	-98,42	146,66	-0,15
SAsr							82,58	186,81	0,09
R^2		0,326***			0,339			0,343	
ΔR^2					0,013			0,004	

[†]Inverse transformed; [††]1/square root (9-12 years) and square transformed (13-16 years).

B, unstandardized coefficients; *SE B*, standard error of *B*; *β*, standardized coefficients.

ΔR^2, R^2 change; height and weight are standardized estimates; SAsr, standardized residuals of skeletal age on chronological age.

SAsr × height, interaction of SAsr with height; SAsr × height × weight, interaction of SAsr with height and weight; variables entered the model at step 1: height and height × weight; variables entered the model at step 2: SAsr × height, and SAsr × height × weight; variables entered the model at step 3: SAsr.

$^{**}p$ <0.01; $^{***}p$ <0.001.

Table S7. Hierarchical regression analysis for body size and standardised residuals of Fels skeletal age on chronological age in the yoyo endurance run.

	Yoyo Endurance Run, meters								
	Step 1			Step 2			Step 3		
Variable	*B*	*SE B*	*β*	*B*	*SE B*	*β*	*B*	*SE B*	*β*
13-16 years (n = 34)									
Height	-24,23	94,48	-0,05	-3,94	108,62	-0,01	-22,86	98,20	-0,04
Height × weight	-162,72	81,27	-0,35	-182,05	86,30	-0,40*	-186,09	77,85	-0,41*
SAsr × height				80,87	99,90	0,15	11,26	93,54	0,02
SAsr × height × weight				-26,00	81,69	-0,06	-220,19	101,75	-0,54*
SAsr							358,60	129,61	0,67*
R^2		0,118			0,138			0,323	
ΔR^2					0,020			0,185*	

B, unstandardized coefficients; *SE B*, standard error of *B*; *β*, standardized coefficients.

ΔR^2, R^2 change; height and weight are standardized estimates; SAsr, standardized residuals of skeletal age on chronological age.

SAsr × height, interaction of SAsr with height; SAsr × height × weight, interaction of SAsr with height and weight; variables entered the model at step 1: height and height × weight; variables entered the model at step 2: SAsr × height, and SAsr × height × weight; variables entered the model at step 3: SAsr.

*p <0.05.
